# Comparative Evaluation of the Reticulocyte Hemoglobin Content Assay When Screening for Iron Deficiency in Elderly Anemic Patients

**DOI:** 10.1155/2011/925907

**Published:** 2011-07-27

**Authors:** Torbjörn Karlsson

**Affiliations:** Department of Hematology, Uppsala University Hospital, 751 85 Uppsala, Sweden

## Abstract

The aim of this study was to evaluate sensitivity and specificity for reticulocyte hemoglobin content (CHr) compared to other hematimetric and biochemical iron parameters, in particular, mean corpuscular hemoglobin (MCH), when screening for iron deficiency in elderly anemic patients. Bone marrow staining negative for iron was used as the gold standard criterion for iron deficiency anemia (IDA). Sensitivity and specificity for CHr, soluble transferrin receptor (sTfR), soluble transferrin receptor/log ferritin (TfR-F index), ferritin, MCH, and transferrin saturation were determined. The best cut-off point for CHr was 30.5 pg corresponding to a sensitivity and specificity of 93% and 69% for IDA, respectively. For MCH, a sensitivity of 93% and a specificity of 86%, respectively, correspond to an optimal cut-off of 28.5 pg. Analysis of CHr was not superior to MCH with respect to sensitivity and specificity when screening for IDA in elderly anemic patients.

## 1. Introduction

Iron deficiency anemia is often a challenge to diagnose using noninvasive methods in elderly anemic patients with comorbid conditions such as cancer, infections, or inflammatory diseases, since biochemical iron status is influenced by acute-phase responses [1]. Under these circumstances the best way to discriminate between IDA and anemia induced by an acute-phase response, that is, anemia of chronic disease (ACD), is to perform a bone marrow examination [2]. In contrast to ACD, there is no stainable bone marrow iron in IDA. The erythrocyte has a lifespan of approximately 120 days in the circulation, which means that the parameter MCH is a measure of the of the iron availability for the erythropoiesis during this time period. In contrast to this, CHr gives a *snapshot* of the iron availability for erythropoiesis, since the lifespan of the reticulocyte is just 1-2 days [3]. Thus, a decrease in reticulocyte hemoglobin content precedes a similar decrease in erythrocytes and is an early and reliable indicator of iron deficiency anemia [4, 5]. CHr, not being affected by acute phase responses, is also an early indicator of functional iron deficiency (FID) in patients treated with erythropoietic stimulating agents [6, 7]. Previous studies assessing a role for CHr in diagnosing iron deficiency have not used the lack of stainable bone marrow iron as the gold standard criterion for iron deficiency or if so, only a fraction of the examined patients have been anemic [4–8]. In the study reported here, where the aim was to evaluate a role for CHr in the diagnosis of iron deficiency, all patients examined were anemic and the gold standard criterion for iron deficiency was lack of stainable bone marrow iron.

## 2. Material and Methods

Sixty consecutive newly diagnosed anemic patients (Hb <134 g/L in men and <117 g/L in women), 60 years of age or older admitted to the Department of Medicine, Capio Sankt Görans Hospital, were screened. All procedures were in accordance with the Helsinki Declaration of 1964. Patients receiving iron supplementation or red cell transfusions were excluded from the study. Those with leukemias, myeloma, myelodysplastic syndromes, folate or coboalamin deficiency, and hemolytic anemia were also excluded, whereas patients with lymphomas without bone marrow involvement were included. Of the 60 screened patients, 6 (10%) were excluded. This observational study was performed on left-over blood and bone marrow samples collected during routine anemia investigation. A complete blood count, biochemical iron status (serum iron, total iron binding capacity [TIBC], transferrin saturation, ferritin), soluble transferrin receptor (sTfR), reticulocyte hemoglobin content (CHr), and bone marrow iron stores were analyzed in every patient. Transferrin saturation is calculated by the formula: serum iron/TIBC. Bone marrow smears were stained by means of the May-Grunwald-Giemsa method, and bone marrow iron stores were investigated using Prussian blue staining. Patients with no stainable bone marrow iron were diagnosed as having iron deficiency anemia (IDA), whereas the diagnosis for those with stainable iron was anemia of chronic disease (ACD). Complete blood counts and CHr analysis were performed using the ADVIA 2120 analyzer (Siemens Diagnostics, Deerfield, IL, USA). C-reactive protein (CRP), iron, and TIBC were investigated by means of ADVIA 2400 and sTfR using BN ProSpec (Siemens Diagnostics, Deerfield, IL, USA). Ferritin was analyzed using ADVIA Centaur XP (Siemens Diagnostics, Deerfield, IL, USA). All reagents were from Siemens Diagnostics except for the TIBC assay, which was from Dakopatts (Dakopatts, Dorchester, UK). In our laboratory reference values for mean corpuscular volume (MCV) are 82–98 fL, mean corpuscular hemoglobin (MCH ) 27–33 pg, iron 9–34 *μ*mol/L, TIBC 47–80 *μ*mol/L, transferrin saturation 15–60% for men and 10–50% for women, ferritin 20–375 *μ*g/L for men and 7–120 *μ*g/L for women, CRP< 5 mg/L, soluble transferrin receptor 0.8–1.7 mg/L, and CHr 24–36 pg. All analyses were performed at the Department of Clinical Chemistry, Capio Sankt Görans Hospital, except for the bone marrow analyses which were performed at the Department of Pathology, Capio Sankt Görans Hospital. Statistical and Receiver Operation Characteristics (ROCs) curve analyses were performed using the SigmaPlot 11 software package (Systat Software, San Jose, CA, USA). Quantitative variables were expressed as means ± standard deviations. The Student's *t* test or Mann-Whitney rank sum test was used to compare the variables between the two groups. A *P* value less than 0.05 was considered statistically significant.

## 3. Results and Discussion

Of the 54 patients studied, 14 (26%) were iron deplete as determined by the absence of stainable bone marrow iron, whereas 40 (74%) were iron replete (Table 1). The dominant clinical diagnoses in the IDA group was benign gastrointestinal hemorrhage (57%) and gastrointestinal hemorrhage with no source of blood loss identified (29%). In the ACD group, the most common diagnoses were infections (21%), cancer (18%), liver disease (18%), fever of unknown origin (13%), and giant cell arteritis (13%). There was no significant difference in age between the two groups, with mean age of 76 and 79 years in the IDA and ACD groups, respectively (Table 1). The mean values of Hb, CHr, MCV, MCH, iron, transferrin saturation, and ferritin were significantly lower in the IDA group compared to the ACD group (Table 1). Soluble transferrin receptor and TIBC were significantly higher in the IDA group, whereas there was no difference for CRP (Table 1). The best cut-off point for CHr was 30.5 pg, corresponding to a sensitivity of 93% and a specificity of 69% for IDA, respectively. For MCH, a sensitivity of 93% and a specificity of 86%, respectively, corresponds to an optimal cut-off of 28.5 pg. Sensitivity, specificity, cut-off points, and ROC area data for CHr, MCH, ferritin, transferrin saturation, sTfR, and TfR-F Index are presented in Table 2. 

The discrimination between IDA and ACD is seldom straightforward in elderly hospitalized anemic patients suffering from pathologic conditions such as cancer, infections, or inflammatory diseases, since biochemical iron status is influenced by acute-phase responses [1]. Several authors [4, 9–11] have suggested different noninvasive tests or combinations of tests, such as CHr, sTfR, and TfR-F Index as alternatives to bone marrow iron staining for discrimination between IDA and ACD. The aim of this study was to evaluate sensitivity and specificity for CHr compared to other hematimetric and biochemical iron tests, in particular MCH, when screening for iron deficiency in elderly hospitalized anemic patients. A comparison between hemoglobin content in reticulocytes (CHr) and erythrocytes (MCH) is especially interesting since the content of reticulocytes reflects iron availability for erythropoiesis during the last 1-2 days in contrast to the latter, the content of which reflects bone marrow iron availability for the entire lifespan of the erythrocyte, that is, 120 days. The results presented here suggest that the CHr assay is not superior to analysis of MCH, ferritin, sTfR, or the TfR-F Index with respect to sensitivity and specificity when screening for iron deficiency in a population of elderly hospitalized anemic patients. Receiver Operating Characteristics analyses provided the highest AUC^ROC^  value for the TfR-F Index, which is in agreement with previously published data [10, 12]. The iron-deficient patients in this study were all absolute iron depleted as determined by bone marrow iron staining, and it is therefore conceivable that the CHr analysis is superior to MCH in earlier stages of iron deficiency or in FID. Other studies have shown higher CHr sensitivity and specificity for iron deficiency anemia or that CHr is superior to MCH analysis when screening for IDA [6, 8, 13]. This study is limited by the relatively low number of patients investigated compared to the other studies discussed here [6, 8, 13]. Furthermore, in contrast to the study by C. Thomas and L. Thomas [11], the group of patients with pure IDA was not analyzed separately from those with IDA complicated by ACD, which could have influenced the results. Other possible explanations for these conflicting data could be that the populations studied were different from the one investigated in this study or that only one of them used the absence of bone marrow iron as the gold standard criterion for IDA.

## 4. Conclusion

Analysis of reticulocyte hemoglobin content is not superior to analysis of the hemoglobin content of the mature erythrocyte (MCH) when screening for iron deficiency in elderly anemic hospitalized patients.

## Figures and Tables

**Table 1:** Comparison of hematimetric and biochemical iron parameters and CRP between patients with IDA and ACD. Data are expressed as means ± SD. IDA, iron deficiency anemia; ACD: anemia of chronic Disease; CHr: reticulocyte hemoglobin content; MCH: mean corpuscular hemoglobin; MCV: mean corpuscular volume; TIBC: total iron binding capacity; TSAT: transferrin saturation, sTfR: soluble transferrin receptor; TfR-F Index, sTfR/log Ferritin; CRP, C-Reactive Protein.

Variable	IDA	ACD	*P*
Age (years)	76 ± 6	79 ± 9	0.291
Hb (g/L)	83 ± 23	100 ± 13	0.012
CHr (pg)	25.9 ± 3.9	31.2 ± 4.2	<0.001
MCH (pg)	24.6 ± 4.3	30.5 ± 3.3	<0.001
MCV (fL)	80.2 ± 10.4	94.3 ± 9.8	<0.001
Iron (*μ*mol/L)	3.8 ± 2.9	9.4 ± 8.7	<0.01
TIBC (*μ*mol/L)	75.3 ± 16.8	45.4 ± 17.2	<0.01
TSAT (%)	8.5 ± 13.2	22.7 ± 2.2	<0.001
sTfR (mg/L)	3.45 ± 1.67	1.41 ± 0.56	<0.001
Ferritin (*μ*g/L)	14 ± 10	439 ± 416	<0.001
TfR-F Index	3.62 ± 3.21	0.69 ± 0.38	<0.001
CRP (mg/L)	20 ± 25	64 ± 33	0.102

**Table 2:** Test characteristics based on optimal Cut-off Values for IDA Screening Determined by ROC Curve Analysis. Abbreviations: CHr, Reticulocyte Hemoglobin Content; MCH, Mean Corpuscular Hemoglobin; TSAT, Transferrin Saturation; sTfR, Soluble Transferrin Receptor; TfR-F Index, sTfR/log Ferritin; IDA, Iron Deficiency Anemia; ROC, Receiver Operation Characteristics.

Variable	Sensitivity (%)	Specificity (%)	Cut-off	ROC area
CHr (pg)	93	69	30.5	0.84
MCH (pg)	93	86	28.5	0.91
TSAT (%)	93	57	12	0.84
sTfR (mg/L)	86	89	2.0	0.92
Ferritin (*μ*g/L)	87	95	30	0.92
TfR-F Index	92	94	1.49	0.97
